# Outcomes After Thrombectomy for Acute Ischemic Stroke Related to Type of Stent Retriever; a MR CLEAN Registry Study

**DOI:** 10.1007/s00270-025-04048-0

**Published:** 2025-06-09

**Authors:** Robrecht R. M. M. Knapen, Robert-Jan B. Goldhoorn, Jan Albert Vos, Bart J. Emmer, Maarten Uyttenboogaart, Jeannette Hofmeijer, Wouter J. Schonewille, Charles B. Majoie, Yvo B. W. E. M. Roos, Aad van der Lugt, Diederik W. J. van Dippel, Hester F. Lingsma, Christiaan van der Leij, Robert J. van Oostenbrugge, Wim H. van Zwam, MR CLEAN Registry study, Diederik W. J. Dippel, Aad van der Lugt, Charles B. L. M. Majoie, Yvo B. W. E. M. Roos, Robert J. van Oostenbrugge, Wim H. van Zwam, Jelis Boiten, Jan Albert Vos, Ivo G. H. Jansen, Maxim J. H. L. Mulder, Robert- Jan B. Goldhoorn, Kars C. J. Compagne, Manon Kappelhof, Josje Brouwer, Sanne J.den Hartog, Wouter H. Hinsenveld, Lotte van den Heuvel, Diederik W. J. Dippel, Bob Roozenbeek, Aad van der Lugt, Pieter Jan van Doormaal, Charles B. L. M. Majoie, Yvo B. W. E. M. Roos, Bart J. Emmer, Jonathan M. Coutinho, Wouter J. Schonewille, Jan Albert Vos, Marieke J. H. Wermer, Marianne A. A. van Walderveen, Adriaan C. G. M. van Es, Julie Staals, Robert J. van Oostenbrugge, Wim H. van Zwam, Jeannette Hofmeijer, Jasper M. Martens, Geert J. Lycklama à Nijeholt, Jelis Boiten, Sebastiaan F. de Bruijn, Lukas C. van Dijk, H. Bart van der Worp, Rob H. Lo, Ewoud J. van Dijk, Hieronymus D. Boogaarts, J. de Vries, Paul L. M. de Kort, Julia van Tuijl, Issam Boukrab, Jo P. Peluso, Puck Fransen, Jan S. P. van den Berg, Heleen M.den Hertog, Boudewijn A. A. M. van Hasselt, Leo A. M. Aerden, René J. Dallinga, Maarten Uyttenboogaart, Omid Eschgi, Reinoud P. H. Bokkers, Tobien H. C. M. L. Schreuder, Roel J. J. Heijboer, Koos Keizer, Rob A. R. Gons, Lonneke S. F. Yo, Emiel J. C. Sturm, Tomas Bulut, Paul J. A. M. Brouwers, Anouk D. Rozeman, Otto Elgersma, Michel J. M. Remmers, Thijs E. A. M. de Jong, Charles B. L. M. Majoie, Wim H. van Zwam, Aad van der Lugt, Geert J. Lycklama à Nijeholt, Marianne A. A. van Walderveen, Marieke E. S. Sprengers, Sjoerd F. M. Jenniskens, René van den Berg, Albert J. Yoo, Ludo F. M. Beenen, Alida A. Postma, Stefan D. Roosendaal, Bas F. W. van der Kallen, Ido R. van den Wijngaard, Adriaan C. G. M. van Es, Bart J. Emmer, Jasper M. Martens, Lonneke S. F. Yo, Jan Albert Vos, Joost Bot, Pieter-Jan van Doormaal, Anton Meijer, Elyas Ghariq, Reinoud P. H. Bokkers, Marc P. van Proosdij, G. Menno Krietemeijer, Jo P. Peluso, Hieronymus D. Boogaarts, Rob Lo, Wouter Dinkelaar, Auke P. A. Appelman, Bas Hammer, Sjoert Pegge, Anouk van der Hoorn, Saman Vinke, Sandra Cornelissen, Christiaan van der Leij, Rutger Brans, Jeanette Bakker, Maarten Uyttenboogaart, Miou Koopman, Lucas Smagge, Olvert A. Berkhemer, Jeroen Markenstein, Eef Hendriks, Patrick Brouwer, Dick Gerrits, Diederik W. J. Dippel, Aad van der Lugt, Charles B. L. M. Majoie, Yvo B. W. E. M. Roos, Robert J. van Oostenbrugge, Wim H. van Zwam, Geert J. Lycklama à Nijeholt, Jelis Boiten, Jan Albert Vos, Wouter J. Schonewille, Jeannette Hofmeijer, Jasper M. Martens, H. Bart van der Worp, Rob H. Lo, Robert J. van Oostenbrugge, Jeannette Hofmeijer, H. Zwenneke Flach, Hester F. Lingsma, Naziha el Ghannouti, Martin Sterrenberg, Wilma Pellikaan, Rita Sprengers, Marjan Elfrink, Michelle Simons, Marjolein Vossers, Joke de Meris, Tamara Vermeulen, Annet Geerlings, Gina van Vemde, Tiny Simons, Gert Messchendorp, Nynke Nicolaij, Hester Bongenaar, Karin Bodde, Sandra Kleijn, Jasmijn Lodico, Hanneke Droste, Maureen Wollaert, Sabrina Verheesen, D. Jeurrissen, Erna Bos, Yvonne Drabbe, Michelle Sandiman, Nicoline Aaldering, Berber Zweedijk, Jocova Vervoort, Eva Ponjee, Sharon Romviel, Karin Kanselaar, Denn Barning, Laurine van der Steen, Esmee Venema, Vicky Chalos, Ralph R. Geuskens, Tim van Straaten, Saliha Ergezen, Roger R. M. Harmsma, Daan Muijres, Anouk de Jong, Olvert A. Berkhemer, Anna M. M. Boers, J. Huguet, P. F. C. Groot, Marieke A. Mens, Katinka R. van Kranendonk, Kilian M. Treurniet, Manon L. Tolhuisen, Heitor Alves, Annick J. Weterings, Eleonora L. F. Kirkels, Eva J. H. F. Voogd, Lieve M. Schupp, Sabine L. Collette, Adrien E. D. Groot, Natalie E. LeCouffe, Praneeta R. Konduri, Haryadi Prasetya, Nerea Arrarte-Terreros, Lucas A. Ramos, Nikki Boodt, Anne F. A. V. Pirson, Agnetha A. E. Bruggeman, Nadinda A. M. van der Ende, Rabia Deniz, Susanne G. H. Olthuis, Floor Pinckaers

**Affiliations:** 1 Department of Radiology & Nuclear Medicine, Maastricht University Medical Center, Maastricht, The Netherlands; 2 School for Cardiovascular Diseases Maastricht (CARIM), Maastricht University, Maastricht, The Netherlands; 3 Department of Neurology, Haaglanden Medical Center, The Hague, The Netherlands; 4 Department of Radiology, Sint Antonius Hospital, Nieuwegein, The Netherlands; 5 Department of Radiology and Nuclear Medicine, Amsterdam UMC, Location University of Amsterdam, Amsterdam, The Netherlands; 6 Department of Neurology and Medical Imaging Center, University Medical Center Groningen, University of Groningen, Groningen, The Netherlands; 7 Department of Neurology, Rijnstate Hospital, Arnhem, The Netherlands; 8 Department of Neurology, Sint Antonius Hospital, Nieuwegein, The Netherlands; 9 Department of Neurology, Amsterdam University Medical Center, University of Amsterdam, Amsterdam, The Netherlands; 10 Department of Radiology and Nuclear Medicine, Erasmus Medical Center, University Medical Center, Rotterdam, The Netherlands; 11 Department of Neurology, Erasmus Medical Center, University Medical Center, Rotterdam, The Netherlands; 12 Department of Medical Decision Making, Erasmus Medical Center, University Medical Center, Rotterdam, The Netherlands; 13 Department of Neurology, Maastricht University Medical Center+, Maastricht, The Netherlands; 14 Radiology, Erasmus MC University Medical Center, Rotterdam, The Netherlands; 15 Department of Radiology and Nuclear Medicine, Amsterdam UMC, Location University of Amsterdam, Amsterdam, The Netherlands; 16 Neurology, Amsterdam UMC, Location University of Amsterdam, Amsterdam, The Netherlands; 17 Department of Neurology, School for Cardiovascular Diseases, Maastricht University Medical Center, Maastricht, The Netherlands; 18 Radiology & Nuclear Medicine, School for Cardiovascular Diseases, Maastricht University Medical Center, Maastricht, The Netherlands; 19 Department of Neurology, Sint Antonius Hospital, Nieuwegein, The Netherlands; 20 Sint Antonius Hospital, Nieuwegein, The Netherlands; 21 Department of Neurology, Leiden University Medical Center, Leiden, The Netherlands; 22 Radiology, Leiden University Medical Center, Leiden, The Netherlands; 23 Department of Neurology, Rijnstate Hospital, Arnhem, The Netherlands; 24 Rijnstate Hospital, Arnhem, The Netherlands; 25 Haaglanden MC, The Hague, The Netherlands; 26 Haaglanden MC, The Hague, The Netherlands; 27 Department of Neurology, HAGA Hospital, The Hague, The Netherlands; 28 HAGA Hospital, The Hague, The Netherlands; 29 Department of Neurology, University Medical Center Utrecht, Utrecht, The Netherlands; 30 Radiology, University Medical Center Utrecht, Utrecht, The Netherlands; 31 Department of Neurology, Radboud University Medical Center, Nijmegen, The Netherlands; 32 Radboud University Medical Center, Nijmegen, The Netherlands; 33 Department of Neurology, Elisabeth-TweeSteden Ziekenhuis, Tilburg, The Netherlands; 34 Department of Neurology, Isala Klinieken, Zwolle, The Netherlands; 35 Isala Klinieken, Zwolle, The Netherlands; 36 Department of Neurology, Reinier de Graaf Gasthuis, Delft, The Netherlands; 37 Reinier de Graaf Gasthuis, Delft, The Netherlands; 38 Elisabeth-TweeSteden Ziekenhuis, Tilburg, The Netherlands; 39 Radboud University Medical Center, Nijmegen, The Netherlands; 40 Department of Neurology, University Medical Center Groningen, Groningen, The Netherlands; 41 Radiology, University Medical Center Groningen, Groningen, The Netherlands; 42 Department of Neurology, Zuyderland Medical Center, Heerlen, The Netherlands; 43 Zuyderland Medical Center, Heerlen, The Netherlands; 44 Department of Neurology, Catharina Hospital, Eindhoven, The Netherlands; 45 Catharina Hospital, Eindhoven, The Netherlands; 46 Department of Neurology, Medisch Spectrum Twente, Enschede, The Netherlands; 47 Medisch Spectrum Twente, Enschede, The Netherlands; 48 Department of Radiology, Amsterdam UMC, Vrije Universiteit Van Amsterdam, Amsterdam, The Netherlands; 49 Department of Radiology, Noordwest Ziekenhuisgroep, Alkmaar, The Netherlands; 50 Department of Radiology, Texas Stroke Institute, Texas, USA; 51 Biomedical Engineering & Physics, Amsterdam UMC, Location University of Amsterdam, Amsterdam, The Netherlands; 52 Public Health, Erasmus MC University Medical Center, Rotterdam, The Netherlands; 53 Albert Schweitzer Hospital, Dordrecht, The Netherlands; 54 Department of Neurology, Albert Schweitzer Hospital, Dordrecht, The Netherlands; 55 Department of Neurology, Amphia Hospital, Breda, The Netherlands; 56 Maastricht (CARIM), MHeNs School for Mental Health and Neuroscience, Maastricht, The Netherlands; 57 MHeNs School for Mental Health and Neuroscience, Maastricht, The Netherlands; 58 Radiology, Amphia Hospital, Breda, The Netherlands; 59 Deventer Hospital, Medisch Spectrum Twente, Enschede, The Netherlands

**Keywords:** Stroke, Endovascular treatment (EVT), Stent retriever (SR), Thrombectomy

## Abstract

**Purpose:**

Endovascular treatment (EVT) with a stent retriever is known to be effective and safe in patients with acute ischemic stroke due to large vessel occlusion. We aimed to compare the most used stent retrievers in a nationwide registry of EVT-treated stroke patients (MR CLEAN Registry).

**Methods:**

Patients with ischemic stroke due to large vessel occlusion, treated with stent retriever thrombectomy (each stent retriever with at least 100 EVTs) as first-line technique in the MR CLEAN Registry, were included. The primary outcome was the modified Rankin Scale (mRS) score at 90-day follow-up. Secondary outcomes included reperfusion (expanded Treatment In Cerebral Infarction [eTICI]), mortality at 90 days, symptomatic intracranial hemorrhage, National Institutes of Health Stroke Scale (NIHSS) score between 24 and 48 h post-EVT, and procedure time. With multivariable regression analyses, we calculated odds ratios (OR) and β-estimates to compare outcomes between the most frequently used stent retrievers, with adjustments for predefined variables. One subgroup analysis focused on the effect of the stent retriever on outcomes in M1 occlusions.

**Results:**

Trevo (Stryker) was the most frequently used stent retriever (n = 1541, 70%). Other types were Solitaire (n = 301, 14%) (Medtronic), Embotrap (n = 255, 11%) (Cerenovus; Johnson&Johnson), and Revive (n = 103, 5.2%) (Cerenovus; Johnson&Johnson).

There was a slightly, but statistically significant, higher 90-day mRS score (adjusted common [ac]OR: 0.75, 95%CI: 0.57–0.99) and mortality rate (aOR: 1.77, 95%CI: 1.16–2.68) for the Solitaire and longer procedure times for the Revive stent (mean: 67.6 versus 58.9 min; adjusted β-estimate: 11.6, 95%CI: 2.69–20.6) compared to the Trevo retriever. There were no outcome differences in the M1 subgroup analyses.

**Conclusion:**

Differences in clinical, technical, and safety outcomes after EVT between the Trevo, Solitaire, Embotrap, and Revive stent retrievers were—although statistically significant—small. Treating physicians should use the stent retriever they are used to, and further studies with more strict patient selection should be conducted to validate these results.

**Graphical Abstract:**

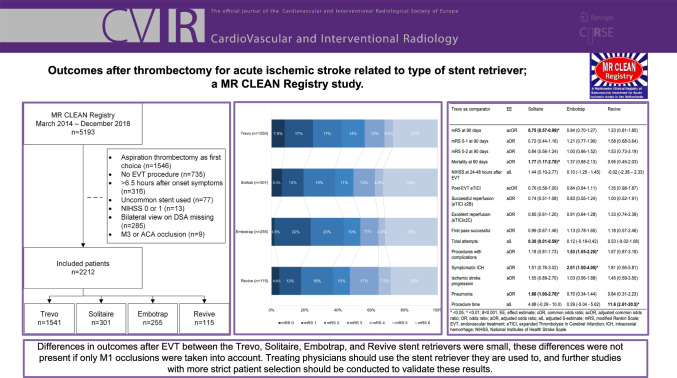

**Supplementary Information:**

The online version contains supplementary material available at 10.1007/s00270-025-04048-0.

## Introduction

Stent retriever thrombectomy has been effective and safe in patients with acute ischemic stroke due to large vessel occlusion (LVO) in the anterior intracranial circulation [1, 2]. Stent retrievers are deployed in the thrombus to capture and then subsequently retrieve it to achieve removal and blood flow restoration [3, 4].

Despite the safety, clinical benefits, and device improvements, overall frequency of complications (e.g., embolization to new territory, dissection, spasm, intracranial hemorrhage, and perforation) during stent retriever thrombectomy is still approximately 15% [5, 6]. As stent retrievers differ in structure and size, differences in performance of each type of stent retriever might be present. Studies comparing technical, clinical, and safety outcomes between different stent retrievers are scarce. Only a few single-center studies compared the Solitaire (Medtronic) with the Trevo stent retriever (Stryker), in which the Trevo stent retriever had higher recanalization rates, shorter procedure times, and less attempts needed; however, these studies compared small groups [7, 8].

Data on individual stent retriever performance may be relevant to gain insight in safety and effectiveness of the treatment. It is known that stent retrievers have different designs (e.g., open-end and close-end baskets, overlapping stent, and dual-layer design), these differences may influence the outcome [9]. We aimed to compare the most used stent retrievers on clinical, technical, and safety outcomes in ischemic stroke patients with large vessel occlusion, treated in the MR CLEAN Registry.

## Method

### Design and Participants

Patients are included from the MR CLEAN Registry, which was a prospective, observational study in 18 endovascular treatment (EVT) performing centers in the Netherlands. The registry started March 2014 and ended December 2018 and included patients treated with EVT for acute ischemic stroke [10]. On reasonable request to the corresponding author, detailed statistical analyses will be made available. Source data will not be made available due to legislative issues on patient privacy. This study was conducted using the STROBE guidelines.

Eligible patients met the following criteria: age ≥ 18 years, groin puncture within 6.5 h after onset symptoms, an anterior circulation occlusion on CT angiography (internal carotid artery [ICA], ICA-terminus [ICA-T], or middle cerebral artery [M1, M2]), and stent retriever as first thrombectomy device. Based on the MR CLEAN Trial, a patient should be treated within 6 h after onset symptoms. However, the time window was extended with 30 min in the MR CLEAN Registry to be compatible with the intended treatment of the trial within six hours. Patients were excluded when direct aspiration only was used as first-line thrombectomy technique, no bilateral view on digital substantiate angiography was performed, and no endovascular treatment was performed. When a brand of stent retriever was rarely (< 100) used in the registry, it was excluded from this study, to minimize differences in experience between stent retrievers.

### Outcomes

The primary outcome was the score on the modified Rankin Scale (mRS) score at 90 days. The mRS score ranges from 0 meaning no disability and 6 death [11]. Secondary outcomes were excellent functional outcome (mRS 0–1), good functional outcome (mRS 0–2), the National Institutes of Health Stroke Scale (NIHSS) score at 24–48 h post-EVT, reperfusion rates measured with the expanded Treatment In Cerebral Ischemia (eTICI) score, procedure duration, total attempts, and first-pass success. Safety outcomes were symptomatic intracranial hemorrhage (sICH), ischemic stroke progression, pneumonia, and total procedures with at least one complication (dissection, embolus in new territory, perforation, distal thrombus, spasm, intracranial hemorrhage, or other).

Reperfusion rates were measured ordinal on the eTICI score, ranging from 0 (no antegrade reperfusion of the affected vascular territory) to grade 3 (complete antegrade reperfusion). Successful reperfusion was defined as a score of ≥ 2B and excellent reperfusion is defined as an eTICI ≥ 2C. A reperfusion grade ≥ 2C after one attempt was considered as first-pass reperfusion. Procedure time was defined as time between groin puncture and reperfusion, when no reperfusion was reached, the time of last digital subtraction angiography (DSA) image was used. An intracranial hemorrhage was considered symptomatic when the patient had neurological deterioration of at least four-point increase on the NIHSS score. The intracranial hemorrhage on follow-up imaging was classified regarding the Heidelberg criteria [12].

### Imaging Assessment

Baseline (non-contrast CT (NCCT) and CT angiography (CTA)), interventional (DSA), and follow-up imaging (NCCT) were assessed by an imaging core laboratory consisting of experienced (neuro)radiologists. The core laboratory was blinded for clinical data. The Alberta Stroke Programme Early CT Score (ASPECTS, a 10-point scale in which points are lost for each of 10 regions affected by ischemia) on NCCT, eTICI on DSA, collateral status (range from no [0] to good [3] collaterals) on CTA, procedural complications on DSA, and intracranial hemorrhage on follow-up imaging were assessed by the core laboratory [13–15].

### Treatment

All patients were treated according to national guidelines. The choice of thrombectomy materials (e.g., balloon guide catheter and stent retriever) and thrombectomy technique was left to the decision of the treating physician. Patients treated with stent retriever thrombectomy combined with direct aspiration thrombectomy as first choice were regarded as treated by stent retriever thrombectomy in this study. The approach for anesthetic management was center-specific and was left to the treating physician and/or anesthesiologist. General anesthesia was defined as the need for airway protection.

### Statistical Analysis

Baseline characteristics are compared between the different stent retrievers and presented with standard statistics, such as mean with standard deviation (SD), median with interquartile range (IQR), and frequency with percentage (%). Multivariable ordinal regression analysis was used to identify differences in outcome between stent retriever types, with the most frequently used stent retriever as comparator. Results were presented as odds ratios (OR) or beta-estimates with corresponding 95% confidence intervals. All analyses were adjusted for: stroke center, time since start MR CLEAN Registry, pre-stroke mRS, age, time between onset symptoms and groin puncture, intravenous thrombolysis (IVT) before EVT, baseline NIHSS, referral from primary stroke center, general anesthesia, the use of a balloon guide catheter, collateral status (continuous), ASPECT score (continuous), and location of occlusion. Variable selection was based on literature and expert opinion. As described, we adjusted for time between start MR CLEAN registry and procedure date, to minimize experience differences between operators. No corrections were made for multiple testing, since we do not interpret results based on p values. All statistics were performed with R (version 4.1.2). The significance level was set at 5%.

### Missing Values

All descriptive analyses were performed with original data. For the regression analysis, missing outcomes and baseline values were replaced by values from multiple imputations with chained equations (MICE) using the *mice* package (version 3.16.0). A predefined set of variables was used for multiple imputations, which was set at 50.

### Subgroup Analysis

Since it may be assumed that different sizes of the stent retrievers will influence the outcome per occlusion location, we predefined a subgroup analysis with only occlusions of the M1 segment of the middle cerebral artery to minimalize this effect. The difference in the effect of stent retriever on outcome in M1 segment occlusions was tested with a likelihood-ratio test with and without interaction term. If the interaction was significant, the effect of stent retriever on outcome in the M1 group was analyzed and presented separately, with the largest stent retriever group as comparator.

## Results

**Fig. 1:**
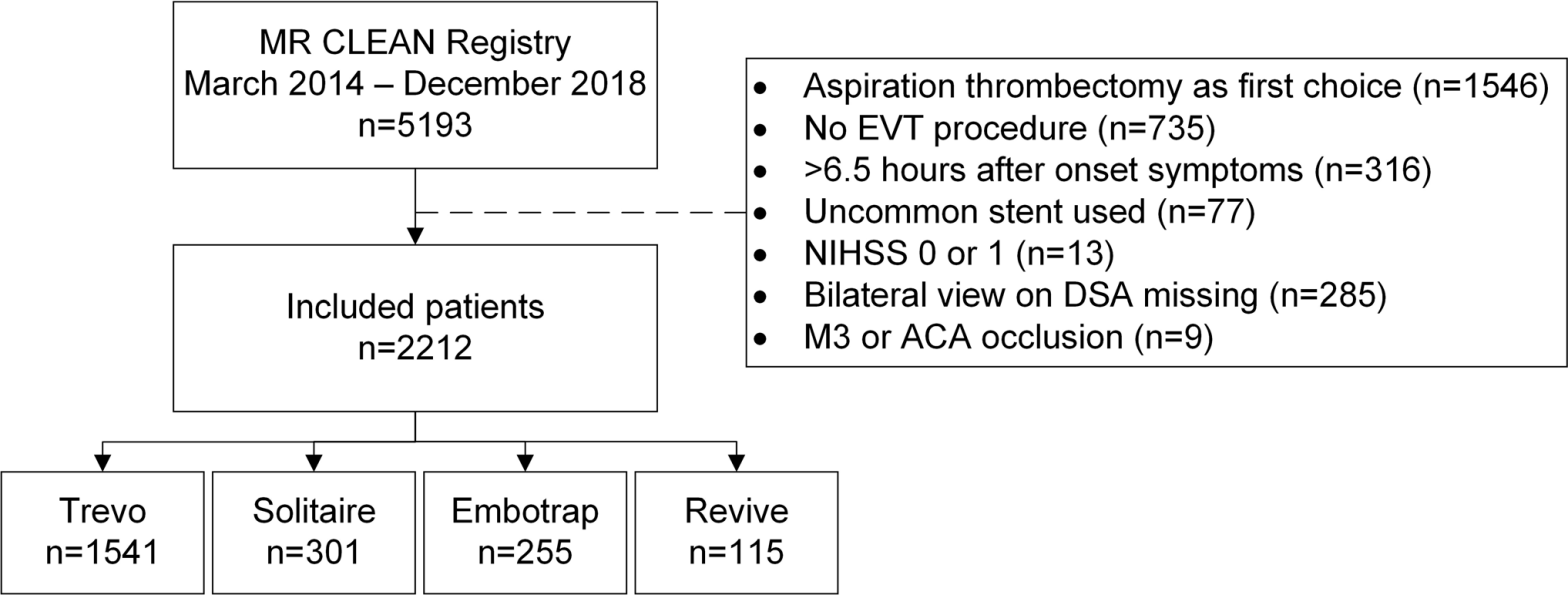
Flowchart of included patients. MR CLEAN, Multicenter collaboration for endovascular treatment of acute ischemic stroke in the Netherlands; EVT, endovascular treatment; NIHSS, National Institutes of Health Stroke Scale

A total of 5193 patients are included in the MR CLEAN Registry (March 2014—December 2018). After applying, the inclusion and exclusion criteria for the present study 2212 patients remained in our analysis (Fig. 1). Trevo stent retriever (Stryker) was the most frequently used (n = 1541; 70%), followed by Solitaire (n = 301; 14%) (Medtronic), Embotrap (n = 255; 12%) (Cerenovus; Johnson&Johnson), and Revive (n = 115; 5.2%) (Cerenovus; Johnson&Johnson) (Figure S1). The mRS was missing in 197 cases (8.9%) and NIHSS in 99 cases (4.5%) (Table 1). At baseline, the usage of a balloon guide catheter, time between onset and groin puncture, occlusion location, transferred to stroke center, and thrombectomy under general anesthesia differed between the stent retrievers (Table 1).

**Table 1:** Patients’ baseline characteristics

	Trevo n = 1541	Solitaire n = 301	Embotrap n = 255	Revive n = 115	Missing (%)	P value
Age–mean (SD)	70.1 (14)	69.4 (14)	71.3 (14)	70.6 (13)	0.0	0.397
Male sex–n. (%)	834 (54)	138 (46)	128 (50)	61 (53)	0.0	0.057
NIHSS–mean (SD)	15.3 (6.1)	15.5 (6.2)	15.4 (5.4)	15.6 (5.5)	0.5	0.856
Pre-mRS–n. (%)	2.3	0.571				
0	985 (66)	194 (65)	152 (62)	69 (60)		
1	205 (14)	38 (13)	40 (16)	23 (20)		
2	113 (7.5)	29 (9.7)	23 (9.4)	10 (8.7)		
> 2	198 (13)	37 (12)	31 (13)	13 (11)		
IVT–n. (%)	1088 (70)	211 (71)	176 (69)	86 (75)	0.3	0.731
Systolic blood pressure–mean mmHg (SD)	149 (25)	149 (27)	148 (25)	151 (26)	2.3	0.549
Previous ischemic stroke–n. (%)	274 (18)	52 (17)	44 (17)	19 (17)	0.6	0.974
Atrial fibrillation–n. (%)	384 (25)	75 (25)	60 (24)	30 (27)	1.1	0.924
Hypertension–n. (%)	801 (53)	149 (50)	129 (51)	54 (49)	2.0	0.645
Hypercholesterolemia–n. (%)	460 (31)	76 (26)	70 (29)	28 (29)	3.1	0.361
Diabetes mellitus–n. (%)	263 (17)	62 (21)	42 (17)	13 (11)	0.5	0.117
Current smoking–n. (%)	322 (27)	60 (24)	53 (28)	26 (35)	22	0.276
Transferred to stroke center–n. (%)	825 (54)	137 (46)	176 (69)	63 (55)	0.0	**< 0.001**
Occlusion location–n (%)					3.4	**0.018**
ICA	68 (4.6)	16 (5.7)	9 (3.6)	3 (2.7)		
ICA-T	283 (19)	69 (24)	59 (23)	17 (15)		
MCA segment M1	876 (59)	143 (51)	149 (59)	80 (71)		
MCA segment M2	262 (18)	55 (19)	35 (14)	12 (11)		
ASPECT group	2.8	0.187				
0–4–n. (%)	69 (4.6)	14 (4.8)	9 (3.6)	6 (5.5)		
5–7–n. (%)	284 (19)	46 (16)	55 (22)	26 (24)		
8–10–n. (%)	1144 (76)	234 (80)	186 (74)	78 (71)		
Collaterals	4.7	0.140				
Grade 0–n. (%)	80 (5.5)	19 (6.7)	15 (5.9)	5 (4.7)		
Grade 1–n. (%)	533 (36)	102 (36)	100 (39)	34 (32)		
Grade 2–n. (%)	559 (38)	103 (37)	110 (43)	44 (41)		
Grade 3–n. (%)	293 (20)	58 (21)	29 (11)	24 (22)		
Onset to groin–mean minutes (SD)	198 (77)	195 (75)	214 (72)	203 (68)	1.1	**0.012**
Balloon guide catheter–n. (%)	991 (75)	180 (71)	106 (51)	64 (68)	15	**< 0.001**
General anesthesia–n. (%)	219 (15)	87 (30)	110 (43)	22 (20)	2.9	**< 0.001**

SD, standard deviation; NIHSS, National Institutes of Health Stroke Scale; mRS, modified Rankin Scale; IVT, intravenous thrombolysis; ICA, internal carotid artery; ICA-T, internal carotid artery terminus; MCA, middle cerebral artery; ASPECTS, Alberta Stroke Program Early Computed Tomography score; CTA, CT angiography

### Clinical Outcome

**Fig. 2:**
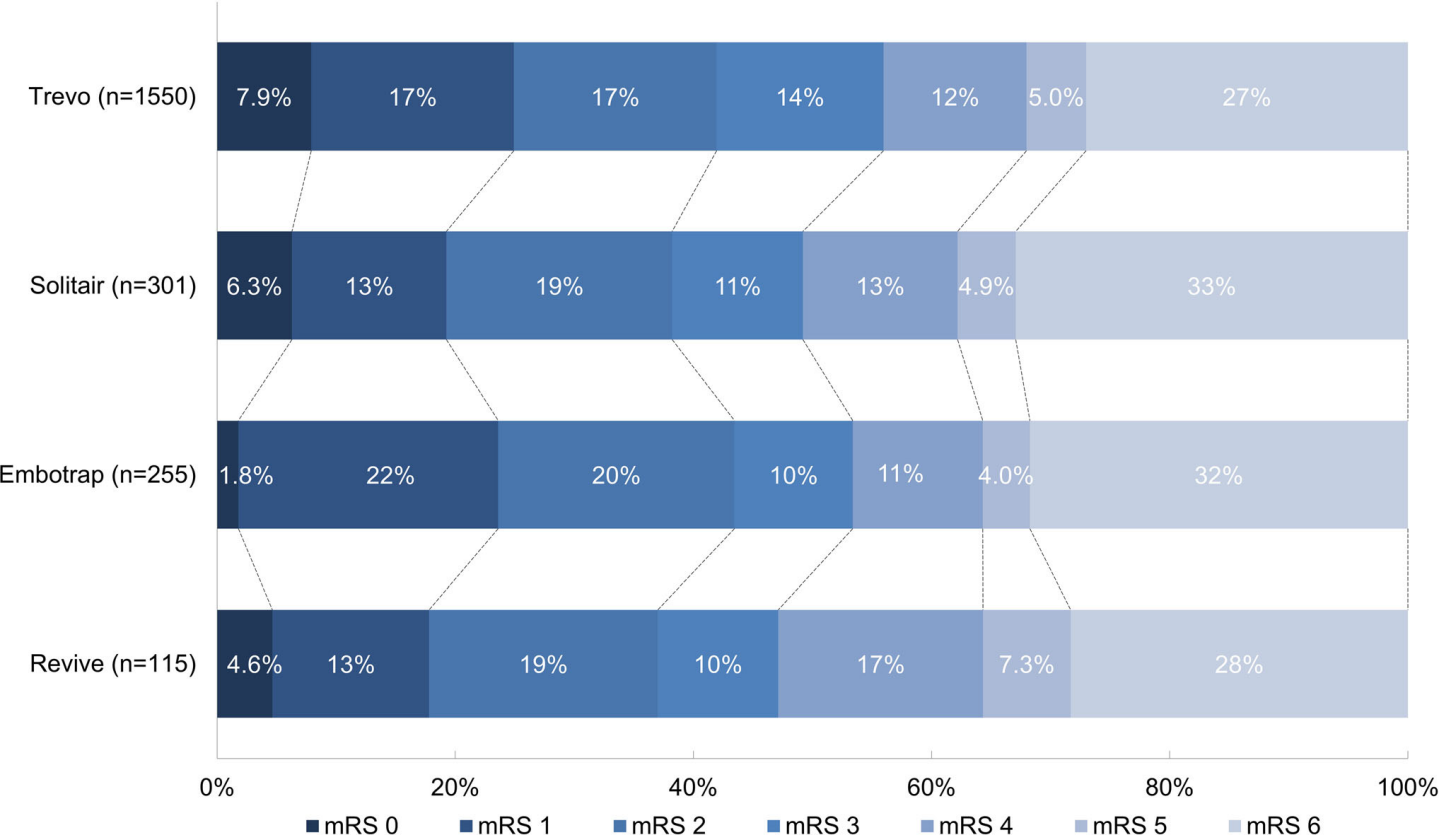
Distribution of the modified Rankin Scale between stent retrievers. mRS, modified Rankin Scale

The mRS score at 90-day follow-up was worse with the Solitaire retriever compared to the Trevo stent retriever (adjusted common [ac]OR: 0.75, 95%CI: 0.57–0.99) (Table 3, Fig. 2). No other differences were seen between the Trevo and other stent retrievers on the primary outcome (Table 3). In the secondary outcomes, patients treated with the Solitaire stent retriever had higher odds of mortality at 90-day follow-up compared to the Trevo stent retriever (33% [n = 94] versus 27% [n = 370], aOR: 1.77, 95%CI: 1.17–2.70) (Table 3). Good functional outcome was not statistically significant different between the groups, in 43% (n = 596) in the Trevo group, in 39% (n = 111) in the Solitaire group, in 44% (n = 100) in the Embotrap group, and in 37% (n = 40) in the Revive group.

### Technical Outcome

No statically significant differences were seen in successful, excellent, and first-pass reperfusion rates among the stent retrievers (Tables 2 and 3). The Solitaire (mean 2.6; aß: 0.30, 95%CI: 0.01–0.59) stent retriever showed higher total attempts compared to the Trevo stent retriever (mean 2.4). The procedure duration was significantly longer in patients treated with the Revive retriever compared to the Trevo (mean 67.6 versus 58.9 min; aß: 11.6, 95%CI: 2.61–20.5) (Table 3). No other differences in procedure time were objectivated.

**Table 2:** Clinical and functional outcomes

	Trevo n = 1541	Solitaire n = 301	Embotrap n = 255	Revive n = 115
mRS at 90 days–n/N. (%)				
0	110/1393 (7.9)	18/288 (6.3)	4/228 (1.8)	5/109 (4.6)
1	242/1393 (17)	38/288 (13)	51/228 (22)	14/109 (13)
2	244/1393 (18)	55/288 (19)	45/228 (20)	21/109 (19)
3	195/1393 (14)	31/288 (11)	23/228 (10)	11/109 (10)
4	162/1393 (12)	36/288 (13)	24/228 (11)	19/109 (17)
5	70/1393 (5.0)	14/288 (4.9)	9/228 (4.0)	8/109 (7.3)
6	370/1393 (27)	94/288 (33)	72/228 (32)	31/109 (28)
mRS 0–1 at 90 days–n/N.(%)	352/1393 (25)	56/288 (20)	55/228 (24)	19/109 (17)
mRS 0–2 at 90 days–n/N.(%)	596/1393 (43)	111/288 (39)	100/228 (44)	40/109 (37)
Mortality at 90 days–n/N. (%)	370/1393 (27)	94/288 (33)	72/228 (32)	31/109 (28)
NIHSS post-intervention–mean (SD)*	10.9 (9.5)	12.0 (11)	11.1 (8.3)	13.1 (8.7)
Successful reperfusion (eTICI ≥ 2B)–n/N. (%)	1155/1506 (77)	213/290 (73)	196/251 (78)	79/114 (69)
Excellent reperfusion (eTICI ≥ 2C)–n/N. (%)	793/1506 (53)	147/290 (51)	140/251 (56)	52/114 (46)
Total attempts–mean (SD)^	2.4 (1.7)	2.6 (2.1)	2.3 (1.6)	2.8 (1.8)
First-pass successful–n/N. (%)	336/1445 (23)	62/287 (22)	76/249 (31)	23/113 (20)
Procedures with complications–n/N. (%)	349/1481 (24)	71/271 (26)	76/253 (30)	39/115 (34)
sICH–n. (%)	71 (4.6)	23 (7.6)	19 (7.5)	8 (7.0)
Ischemic stroke progression–n. (%)	121 (7.9)	29 (9.6)	26 (10)	17 (15)
Pneumonia–n. (%)	155 (10)	40 (13)	14 (4.7)	14 (12)
Procedure time–mean minutes (SD)	58.9 (32)	68.6 (40)	53.1 (30)	67.6 (37)

^*^NIHSS was missing in 99 patients; ^ total attempts were missing in 208 patients; # procedure time was missing in 57 patients

mRS, modified Rankin Scale; eTICI, expanded Thrombolysis In Cerebral Infarction; sICH, symptomatic intracranial hemorrhage; NIHSS, National Institutes of Health Stroke Scale; mRS, modified Rankin Scale; SD, standard deviation

**Table 3:** Regression analyses outcomes

Trevo as comparator		Solitaire	Embotrap	Revive
	EE			
mRS at 90 days	cOR	0.76 (0.61–0.95)*	0.90 (0.71–1.15)	0.76 (0.54–1.06)
	acOR	0.75 (0.57–0.99)*	0.94 (0.70–1.27)	1.23 (0.81–1.85)
mRS 0–1 at 90 days	OR	0.72 (0.53–0.99)*	0.97 (0.71–1.34)	0.62 (0.37–1.03)
	aOR	0.72 (0.44–1.16)	1.21 (0.77–1.90)	1.58 (0.68–3.64)
mRS 0–2 at 90 days	OR	0.84 (0.65–1.09)	1.08 (0.82–1.42)	0.78 (0.52–1.16)
	aOR	0.84 (0.56–1.24)	1.00 (0.66–1.52)	1.53 (0.73–3.19)
Mortality at 90 days	OR	1.37 (1.04–1.79)*	1.21 (0.90–1.63)	1.08 (0.70–1.67)
	aOR	**1.77 (1.17–2.70)^**	1.37 (0.88–2.13)	0.95 (0.45–2.03)
NIHSS at 24–48 h after EVT	ß	0.97 (-0.24–2.17)	0.04 (-1.22–1.30)	2.17 (0.33–4.02)*
	aß	1.44 (0.10–2.77)	0.10 (-1.25—1.45)	-0.02 (-2.38–2.33)
Post-EVT eTICI	OR	0.87 (0.69–1.09)	1.04 (0.82–1.32)	0.76 (0.53–1.07)
	acOR	0.76 (0.58–1.00)	0.84 (0.64–1.11)	1.35 (0.98–1.87)
Successful reperfusion (eTICI ≥ 2B)	OR	0.84 (0.63–1.13)	1.08 (0.78–1.49)	0.69 (0.45–1.04)
	aOR	0.74 (0.51–1.08)	0.83 (0.55–1.24)	1.00 (0.52–1.91)
Excellent reperfusion (eTICI ≥ 2C)	OR	0.93 (0.72–1.20)	1.12 (0.86–1.47)	0.75 (0.51–1.11)
	aOR	0.85 (0.61–1.20)	0.91 (0.64–1.28)	1.33 (0.74–2.39)
First-pass successful	OR	0.94 (0.70–1.28)	1.41 (1.05–1.90)*	0.81 (0.51–1.31)
	aOR	0.99 (0.67–1.46)	1.13 (0.78–1.65)	1.18 (0.57–2.46)
Total attempts	ß	0.14 (-0.09–0.38)	-0.12 (-0.37–0.13)	0.34 (-0.02–0.71)
	aß	0.30 (0.01–0.59)*	0.12 (-0.19–0.42)	0.53 (-0.02–1.08)
Procedures with complications	OR	1.10 (0.82–1.49)	1.39 (1.03–1.86)*	1.66 (1.11–2.49)*
	aOR	1.18 (0.81–1.73)	1.53 (1.05–2.25)*	1.67 (0.87–3.18)
Symptomatic ICH	OR	1.71 (1.05–2.79)*	1.67 (0.99–2.82)	1.55 (0.73–3.30)
	aOR	1.51 (0.76–3.02)	2.01 (1.00–4.05)*	1.81 (0.56–5.81)
Ischemic stroke progression	OR	1.25 (0.82–1.92)	1.33 (0.85–2.08)	2.04 (1.18–3.52)*
	aOR	1.55 (0.89–2.70)	1.03 (0.56–1.88)	1.45 (0.59–3.55)
Pneumonia	OR	1.37 (0.94–1.99)	**0.44 (0.24–0.80)^**	1.24 (0.69–2.22)
	aOR	1.66 (1.00–2.76)*	0.70 (0.34–1.44)	0.84 (0.31–2.23)
Procedure time	ß	**9.94 (5.78–14.1)**^**#**^	**-5.82 (-10.3–-1.36)**^**#**^	8.79 (2.49–15.1)*
	aß	4.88 (-0.29—10.0)	0.29 (-5.04—5.62)	11.6 (2.61–20.5)*

^*^ < 0.05; ^ < 0.01; # < 0.001

EE: effect estimate; cOR: common odds ratio: acOR: adjusted common odds ratio; OR: odds ratio; aOR: adjusted odds ratio; aß: adjusted ß-estimate; mRS: modified Rankin Scale; EVT: endovascular treatment; eTICI: expanded Thrombolysis In Cerebral Infarction; ICH: intracranial hemorrhage; NIHSS: National Institutes of Health Stroke Scale

### Safety Outcomes

Symptomatic ICH did not differ between patients treated with Trevo (n = 71; 4.6%), Solitaire (n = 23; 7.6%), or Revive (n = 8; 7.0%) stent retriever (Tables 2 and 3). The Embotrap (n = 19; 7.5%) had higher changes of sICH rates and procedural complications compared to the Trevo stent retriever (aOR: 2.01, 95%CI: 1.00–4.05, and aOR: 1.53, 95%CI: 1.05–2.25, respectively). The procedural complications are presented in Table S2. Patients treated with Solitaire retriever had higher changes of pneumonia (aOR: 1.66, 95%CI: 1.00–2.76) compared to the Trevo group (Table 3). Ischemic stroke progression did not differ (Tables 2and 3).

### Sensitivity Analysis

A significant interaction effect estimate between the occlusion location and the effect of the different stent retrievers on the mRS at 90 days was found (*P* < 0.001). However, in the group of M1 occlusions, no difference in mRS at 90 days was observed for Solitaire (acOR: 0.77, 95%CI: 0.52–1.13), Embotrap (acOR: 0.97, 95%CI: 0.65–1.46), or the Revive (acOR: 1.59, 95%CI: 0.94–2.69) stent retrievers (Table S1), compared to Trevo. Similarly, no significant differences were seen in excellent and good functional outcome (Table S1).

## Discussion

In this nationwide study, we compared four different stent retrieves regarding clinical, safety, and technical outcomes in patients with acute ischemic stroke due to anterior circulation occlusion.

Our study showed small, but statistically significant, differences in clinical outcome in favor of the Trevo stent retriever, without differences in reperfusion rates. Earlier meta-analysis and studies comparing Trevo and Solitaire showed opposite results with no significant differences in clinical outcome, while the Trevo stent retriever achieved higher reperfusion rates and shorter procedure time in two studies, but not within the meta-analysis [7, 8, 16–18]. A potential reason may be the larger groups and the potential selection bias present in the study arms, as there will be differences in experience with each stent retriever (especially in the Trevo and Solitaire group) and as different sizes of stent retrievers may be used in different occlusion locations. Although we adjusted for the location, influence may still occur. Therefore, we performed a sensitivity analysis with only M1 occlusions in which the differences in clinical outcomes were not observed. Results must, therefore, be interpreted with caution.

The longer procedure times with Revive might be due to the fact that this stent retriever was mainly used in the first years of the MR CLEAN Registry (Figure S1). Although we adjusted for the time between start registry and day of treatment, the experience of the treating physician over time may result in shorter procedure times.

The ARISE II study showed better Embotrap results compared to our results, higher first-pass reperfusion rates (52% vs. 31%), lower mortality rates (9.2% vs. 32%), shorter procedure time (median 35 [IQR:24.0–58.0] versus mean 53.1 [SD:30.0]), and higher functional outcome rates (67% vs 44%) [19]. A possible reason for the differences in outcome may be the first-line thrombectomy technique and the use of a balloon guide catheter. The ARISE II study reported 74% of the patients treated with a balloon guide catheter, while our percentage for the Embotrap group is 51%. It is known that stent retriever thrombectomy alone (without direct aspiration thrombectomy) combined with a balloon guide catheter results in better outcomes than stent retriever thrombectomy alone without a balloon guide catheter [20–22]. In addition, another important explanation is the study population. In the MR CLEAN Registry, all patients who received EVT were included, whereas patients in the ARISE II study were included when strict in- and exclusion criteria were met.

In this study, we observed no differences between Trevo and Solitaire stent retriever concerning the occurrence of sICH, which is similar to previous studies comparing both stent retrievers [8, 16]. Procedural complications and sICH were more often seen in the Embotrap group compared to the Trevo group; however, this did not lead to differences in clinical outcome. Approximately 10% of the patients in our study developed a poststroke pneumonia (PSP). In the Solitaire stent retriever group, more patients suffered from a PSP (13%). Patients treated with the Solitaire stent retriever were treated more under general anesthesia compared to patients treated with the Trevo stent retriever. General anesthesia is a risk factor for PSP, and PSP has been associated with poor outcomes [23–25]. This is a potential added argument why patients treated with Solitaire had higher mortality rates and higher mRS rates at 90 days.

This study contains limitations related to its observational design, including selection bias and free choice of endovascular approach. In our study, we did not take the length or diameter of the stent retriever into account, nor the additional use of a distal access catheter, since these data were not registered properly. These aspects may influence the reperfusion rates [26]. However, the choice of stent retriever size (length and diameter) mainly depends on the location of the occlusion, we tried to minimize this effect to adjust for the occlusion location. Our subgroup analysis of M1 occlusions showed no difference in stent retrievers. Moreover, no differentiation was made between stent retriever-only thrombectomy and the combined thrombectomy technique; however, the ASTER-2 trial and a meta-analysis showed no significant differences in clinical and technical outcome between stent retriever thrombectomy and the combined technique, meaning that this data are still relevant [27, 28]. Additionally, over the years different generation of stent retrievers are developed and this is not taken into account in this study. Therefore, interpretation needs to be done with caution. Further, the experience level of the treating physician with certain stent retrievers may differ over the years, and between the treating physicians, which can influence outcomes. Olthuis et al. showed better outcomes after EVT when interventionist treated more patients in the preceding year. However, less effect on outcome was seen in the total number of procedures an interventionist has performed previously [29]. The treating physicians were free to switch between the stent retrievers, while only the first-choice stent retriever was analyzed. Finally, the proportional use of each stent retriever differs and certain stent retriever brands, including newer generations of stent retrievers, were excluded, but still, every stent retriever was used at least 100 times. Over the years, the use of the Revive stent retriever has become less, whereas the Embotrap is used more, especially since 2016 (Figure S1).

## Conclusion

Differences in outcomes after EVT between the Trevo, Solitaire, Embotrap, and Revive stent retrievers were—although statistically significant—small. These differences were not present if only M1 occlusions were taken into account. Treating physicians should use the stent retriever they are used to, and further studies with more strict patient selection should be conducted to validate these results.

## Supplementary Information

Below is the link to the electronic supplementary material.

Supplementary file1 (DOCX 615 KB)
