# Supplementary material for: Outcomes After Thrombectomy for Acute Ischemic Stroke Related to Type of Stent Retriever; a MR CLEAN Registry Study
Source: Cardiovasc Intervent Radiol. 2025 Jun 9;48(8):1126–39. doi: 10.1007/s00270-025-04048-0 (PMC12325387; doi:10.1007/s00270-025-04048-0)
Supplement: Supplementary file 1 — Supplementary file1 (DOCX 615 KB) [file 270_2025_4048_MOESM1_ESM.docx]

**Supplemental material**

**Figure S1.** Overview of stent retriever use per year in percentages.
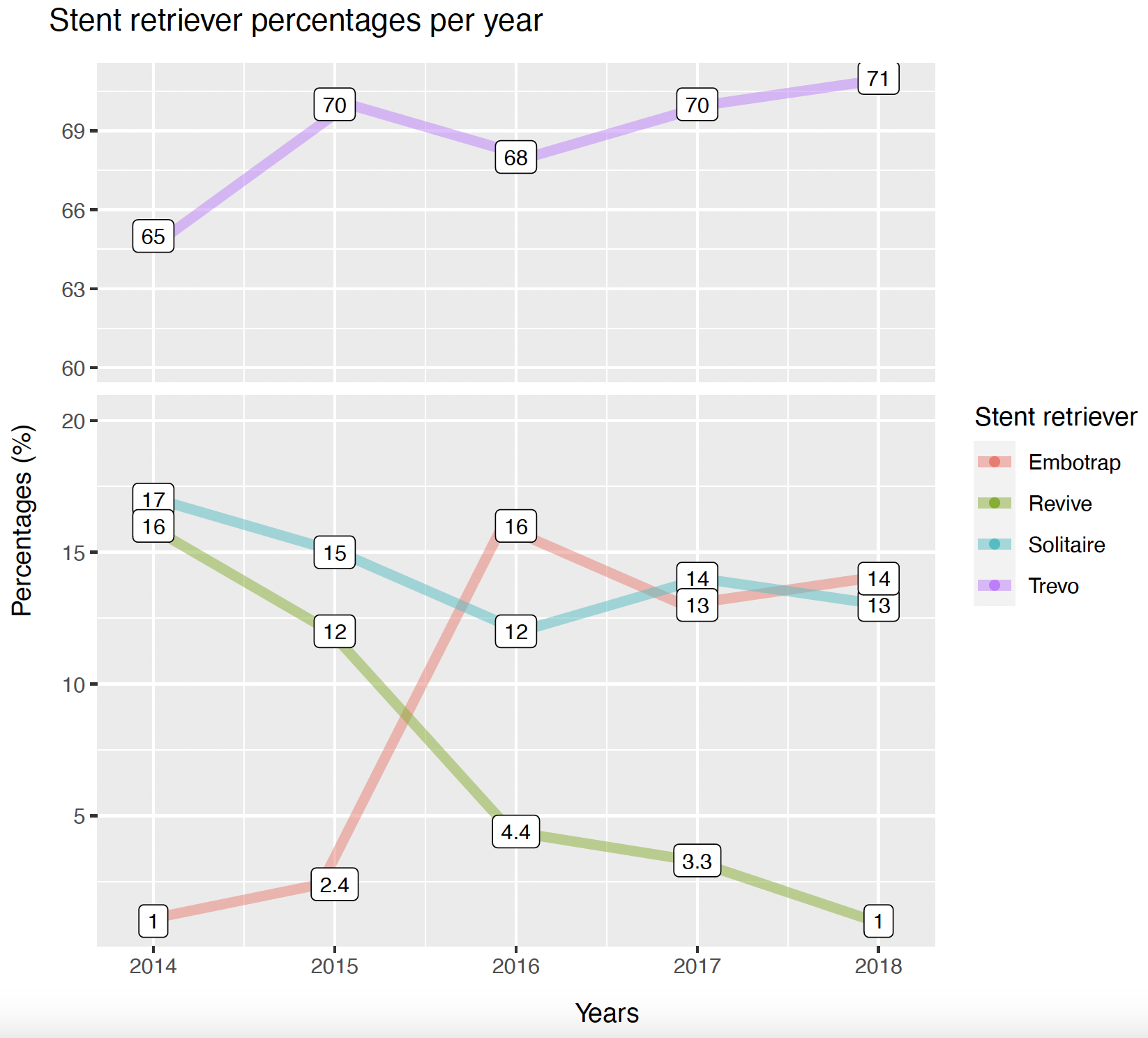


**Table S1.** Sensitivity analysis in patients with an M1 occlusion.

| Trevo as  comparator |  | Solitaire | Embotrap | Revive |
| --- | --- | --- | --- | --- |
|  | EE | | | |
| mRS at 90 days | cOR | 0.74 (0.54-1.03) | 0.89 (0.65-1.22) | 0.73 (0.49-1.09) |
|  | acOR | 0.77 (0.52-1.13) | 0.97 (0.65-1.46) | 1.59 (0.94-2.69) |
| mRS 0-1 at 90 days | OR | **0.72 (0.53-0.99)*** | 0.97 (0.71-1.34) | 0.62 (0.37-1.03) |
|  | aOR | 0.70 (0.43-1.12) | 1.20 (0.77-1.88) | 1.66 (0.72-3.81) |
| mRS 0-2 at 90 days | OR | 0.81 (0.57-1.17) | 0.94 (0.65-1.35) | 0.72 (0.45-1.17) |
|  | aOR | 0.75 (0.42-1.33) | 0.79 (0.45-1.37) | 1.47 (0.55-3.93) |
| Mortality at 90 days | OR | 1.46 (0.99-2.15) | 1.17 (0.78-1.75) | 1.11 (0.65-1.87) |
|  | aOR | 1.66 (0.91-3.02) | 1.28 (0.71-2.31) | 0.61 (0.21-1.73) |

* <0.05; ^ <0.01; #<0.001

mRS, modified Rankin Scale.

**Table S2.** Overview of the procedural complications.

|  | Trevo  n=1541 | Solitaire  n=301 | Embotrap  n=255 | Revive  n=115 |
| --- | --- | --- | --- | --- |
| Procedures with complications – n. (%) | 349 (24) | 71 (26) | 76 (30) | 39 (34) |
| Total complications – n/N. (%) | | | | |
| Dissection | 21/398 (5.3) | 3/78 (3.8) | 6/84 (7.1) | 1/43 (2.3) |
| Embolus in new territory | 81/398 (20) | 12/78 (15) | 14/84 (17) | 9/43 (21) |
| Perforation | 16/398 (4.0) | 2/78 (2.6) | 4/84 (4.8) | 0/43 |
| Distal thrombus | 162/398 (41) | 41/78 (53) | 43/84 (51) | 21/43 (49) |
| Spasm | 97/398 (24) | 17/78 (22) | 14/84 (17) | 11/43 (26) |
| Intracranial hemorrhage | 3/398 (0.8) | 0/78 | 0/84 | 0/43 |
| Other | 18/398 (4.5) | 3/78 (3.8) | 1/84 (1.2) | 1/43 (2.3) |
